# Assessing the Readability of Medical Documents: A Ranking Approach

**DOI:** 10.2196/medinform.8611

**Published:** 2018-03-23

**Authors:** Jiaping Zheng, Hong Yu

**Affiliations:** ^1^ College of Information and Computer Sciences University of Massachusetts Amherst, MA United States; ^2^ Center for Healthcare Organization and Implementation Research Bedford Veterans Affairs Medical Center Bedford, MA United States; ^3^ Department of Computer Science University of Massachusetts Lowell, MA United States; ^4^ Department of Medicine University of Massachusetts Medical School Worcester, MA United States

**Keywords:** electronic health records, readability, comprehension, machine learning

## Abstract

**Background:**

The use of electronic health record (EHR) systems with patient engagement capabilities, including viewing, downloading, and transmitting health information, has recently grown tremendously. However, using these resources to engage patients in managing their own health remains challenging due to the complex and technical nature of the EHR narratives.

**Objective:**

Our objective was to develop a machine learning–based system to assess readability levels of complex documents such as EHR notes.

**Methods:**

We collected difficulty ratings of EHR notes and Wikipedia articles using crowdsourcing from 90 readers. We built a supervised model to assess readability based on relative orders of text difficulty using both surface text features and word embeddings. We evaluated system performance using the Kendall coefficient of concordance against human ratings.

**Results:**

Our system achieved significantly higher concordance (.734) with human annotators than did a baseline using the Flesch-Kincaid Grade Level, a widely adopted readability formula (.531). The improvement was also consistent across different disease topics. This method’s concordance with an individual human user’s ratings was also higher than the concordance between different human annotators (.658).

**Conclusions:**

We explored methods to automatically assess the readability levels of clinical narratives. Our ranking-based system using simple textual features and easy-to-learn word embeddings outperformed a widely used readability formula. Our ranking-based method can predict relative difficulties of medical documents. It is not constrained to a predefined set of readability levels, a common design in many machine learning–based systems. Furthermore, the feature set does not rely on complex processing of the documents. One potential application of our readability ranking is personalization, allowing patients to better accommodate their own background knowledge.

## Introduction

### Background

Research has demonstrated that actively involving patients in the management of their own health can lead to better outcomes, and potentially lower costs [1,2]. Patient engagement [3]—a concept that includes patient activation, and interventions designed to increase activation and promote positive patient behavior—has thus emerged as an important component of strategies to improve health care. A growing body of evidence has accumulated on better health outcomes and care experiences associated with higher engagement. For example, patients with chronic diseases who have high patient activation measure scores are more likely to practice self-management behaviors and report high medication adherence [4]. High patient activation measure scores are also associated with a high likelihood of clinical indicators (eg, hemoglobin A_1c_, high-density lipoprotein, and triglycerides) being in the normal range [1].

The use of electronic health record (EHR) systems with patient engagement capabilities, including viewing, downloading, and transmitting health information, has recently grown tremendously. According to data from the US Office of the National Coordinator for Health Information Technology, the percentage of hospitals that enable patients to electronically view, download, and transmit their health information grew almost 7-fold between 2013 and 2015 [5]. In 2015, 95% of hospitals provided their patients with the ability to view their information.

However, actively engaging patients in the management of their own health remains challenging, despite the evidence of better health care outcomes and potentially lower costs. Access to EHRs by itself is not sufficient to motivate patients to be involved because of the complex and technical nature of the EHR. Patients without training in medicine may struggle to process and understand the information buried in the technical language in EHRs. In fact, materials beyond patients’ reading abilities are widely reported in the literature [6-10]. The lack of explanation that an expert can provide when reading EHR notes may also engender unnecessary anxiety or confusion [11]. Furthermore, many patients have limited health literacy and are not proficient in completing tasks considered essential to successfully navigate the health system and act on health information [12].

Therefore, assessing the difficulty of EHR notes and integrating appropriate educational assistance in EHR systems may make them more accessible for a layperson without professional training in medicine. In this study, we explored methods to automatically assess the readability levels of clinical narratives in EHRs and other complex documents. An accurate assessment of these documents can be used to match patients’ literacy levels, facilitating patient activation and engagement.

### Prior Work

The research community has relied on readability formulas to assess a variety of information materials for patients. Numerous readability metrics have been developed to assess the grade level or the number of years of education needed for a person to understand the content. One of the most widely used in the health domain is the Flesch-Kincaid Grade Level [13] (FKGL), which predicts a grade level based on the average sentence length and the average word length. Other similar metrics are the Simple Measure of Gobbledygook, Gunning Fog Index, Coleman-Liau Index, and New Dale-Chall formula. These metrics rely on the assumption that the longer the words and the sentences are, the more difficult the text is. However, this assumption does not hold for EHR narratives, as sentences are usually short and abbreviations are common.

There were also efforts in the health care domain to develop instruments for medical documents. One measurement proposed by Kim et al [14] compared differences in surface text, syntactic features, and semantic features with a known set of easy and difficult documents and reported normalized scores. Another method for health text was based on a naive Bayes classifier [15]. Those authors collected training documents from blogs, patient education documents, and medical journals. They used vocabularies in these documents as features for the classifier. Both of the methods relied on manually curated documents.

### Goal of This Work

In this work, we considered measuring readability as a ranking task, where the relative difficulty of documents is compared. Readability in the health domain is often measured with formulas developed to ensure that school textbooks are appropriate for children at a particular school grade level [16]. However, obtaining a grade level often is not the ultimate goal. The document’s grade level is usually compared with a person’s educational level or another document’s grade level in order to find appropriate reading materials. The number of years of education has been challenged as a proxy measure for one’s educational experiences when measuring cognitive functions. One study has shown that, in a sample of elderly African Americans, nearly 30% read 3 or more years below their self-reported educational level [17]. Other studies have also advocated the use of reading or literacy ability instead of years of education to account for variance in neuropsychological assessments [18,19].

Therefore, ranking the readability of documents is well suited to applications whose main concern is to match difficulty levels with existing text or to identify easier or more difficult ones, rather than to obtain an absolute score. For example, a patient-facing EHR system may learn from its users’ reactions to infer their reading ability and present appropriate educational materials. Such a system can be personalized for an individual user. A user with limited literacy will only see straightforward materials, whereas higher-quality materials that require higher literacy levels can be presented to an advanced user. This personalization is a first step toward user-centered care. To this end, we developed a machine learning model to compare the relative difficulty of documents using data collected from Amazon Mechanical Turk (AMT) users. A demonstration website is available [20].

## Methods

### Data

We collected difficulty levels on health-related documents from human annotators. We recruited users on AMT (Amazon.com, Inc, Seattle, WA, USA) to read and rate pairs of documents based on their perceived difficulty. We screened AMT users to be from the United States and having an approval rating of at least 95% in prior tasks. Each reader was presented with 20 randomly selected pairs of documents side by side on the computer screen. The readers were requested to rate the readability of the documents on a scale from 1 (easiest to understand) to 10 (most difficult to understand). The setup to show 2 documents helped reduce variation when we assembled the ratings into a complete ranking, as it provided explicit partial ranking, as opposed to implicit order inferred from the difficulty ratings.

The 2 documents in each document pair were of similar length (within a 50-token difference, where a token is a word or term) and comparable difficulty according to FKGL (within 0.5 grade level). We sourced the documents from English Wikipedia articles and deidentified EHR notes written by physicians. The 20 document pairs consisted of 5 pairs of Wikipedia documents, 5 pairs of EHR documents, and 10 pairs of mixed-source documents.

We selected 3 common diseases as topics from the document sources: cancer, diabetes, and hypertension. Wikipedia documents were randomly selected from all article pages up to 3 levels under the disease category page, following the category structure. EHR notes were selected using *International Classification of Diseases, Ninth Revision* codes (140-195 for cancer, 250.00-250.93 for diabetes, and 401.0-401.9 for hypertension). For each disease topic, we collected data from 30 AMT users. In total, 90 AMT users annotated 900 document pairs, with 927 of the documents being unique. Table 1 shows the statistics of the documents annotated by these users.

### Machine Learning System

#### Learning to Rank

We developed a supervised learning system for EHR readability. Traditionally, readability is measured at school grade levels. Formulas that are widely used in the health care domain include the FKGL, Simple Measure of Gobbledygook, Gunning Fog Index, Coleman-Liau Index, and New Dale-Chall formula. They all use a limited number of factors, mostly word and sentence lengths, to estimate a document’s grade level. These simple features, however, are not able to fully capture the complexity of medical documents when used alone as in the formulas. For instance, EHR narratives often contain abbreviations and lists, which are treated as short words and sentences, thus lowering the estimated grade level. However, the abbreviations present a great challenge to a layperson’s understanding [21,22].

In the machine learning community, many systems were developed to classify documents into a predefined set of readability levels. Such systems can include a multitude of features, including lexical, syntactic, and discourse features. These methods are nevertheless constrained in the granularity that they can estimate, since the predefined difficulty levels are often limited.

In our work, we approached readability as a ranking problem, in which the difficulty levels between documents are compared. This approach overcomes the problems in both the traditional formulas and the classification methods: we are not solely reliant on word and sentence lengths as in the formulas, and our approach can order readability levels for a set of documents.

We trained our ranking system using a pairwise approach. From each user’s documents, we generated a training example from any 2 documents that were assigned different difficulty levels.

**Figure 1:**
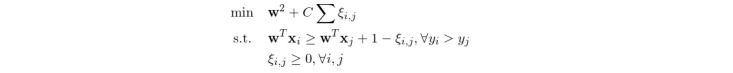
The primal form of pairwise ranking.

A support vector machine (SVM) model was learned from the pairwise comparisons of AMT users’ assigned document difficulty levels using the SVM^rank^ package [23]. SVM models normally optimize a hinge loss function based on a binary label for every training example. In the pairwise scenario, the objective is to minimize the number of discordant pairs—that is, pairs that are ordered incorrectly with respect to the true order. More formally, given a set of training examples {(**x**_i_, y_i_)}, the primal form of the problem is as the equation in Figure 1 shows, where **w** is the weight vector, *C* parameterizes the trade-off between training error and margin size, and ξ is slack variables. Rearranging the first constraint, **w**^T^(**x**_i_–**x**_j_)>1–ξ_i,j_, which is equivalent to a classic SVM problem on the modified input vectors **x**′= **x**_i_–**x**_j_. Therefore, a binary classification SVM optimizer can be used to solve the problem.

In our dataset, we generated pairwise difference vectors **x**′ from each AMT user’s ratings. The difference vectors were not generated from different users because ratings across users may not form a consistent ranking, as those from a single user do. For example, a vector was generated from 2 documents, A and B, by 1 user, but not from 2 documents from different users.

**Table 1:** Statistics of documents annotated by readers.

Source and disease		Documents (n)	Sentences (n)	Tokens^a^ (n)
**Wikipedia**				
	Cancer	215	2510	46,349
	Diabetes	74	1352	33,402
	Hypertension	85	2007	45,440
**EHR^b^** **notes**				
	Cancer	127	2067	37,830
	Diabetes	195	6335	81,085
	Hypertension	231	6594	90,784
Total		927	20,865	334,890

^a^A token is, loosely, a word or term.

^b^EHR: electronic health record.

#### Features

We employed several types of features, including those from traditional readability formulas. We included average words per sentence, average syllables per word from the FKGL formula, proportion of polysyllabic words (words with more than 3 syllables) from the Gunning Fog Index, and percentage of difficult words from the New Dale-Chall formula. Although these formulas do not correlate well with human perceptions of difficulty [24], these word length–based features are useful at capturing some longer medical jargon (eg, Huntington disease). There is also evidence that the perceived difficulty of a word is correlated with its length [25]. We also included word frequency obtained from the Wikipedia documents and EHR notes, since common words have been found likely to be perceived as easier to understand [25]. We grouped the frequencies into 10 bins and used the percentage of words in each bin as features. Additional features included document length measured in words and sentences. Long documents require more cognitive processing to comprehend, which might translate to higher perceived difficulty. Lastly, we captured language patterns using 2 word embeddings learned separately from Wikipedia documents and deidentified EHR notes. We used Word2vec [26] to learn a 200-dimensional skip-gram embedding.

## Results

### System Performance

We split the annotated data three ways, into training (60%), development (20%), and test (20%) sets. The 3 disease topics were stratified in the split. Hyperparameters were optimized on the development set. We obtained final test results from a model trained using the optimized parameters.

We evaluated our system using the Kendall coefficient of concordance (*W*) [27], a statistic that measures the agreement between rankings from multiple raters. The coefficient aggregates the ranks assigned to each item from all raters and measures the variance. The variance is then normalized to be between 0 and 1. Higher values represent a high level of concordance. In our experiments, for each AMT user, we ordered his or her documents by their assigned difficulty levels and calculated *W* with the order generated from our system prediction. We then averaged the *W* coefficients of all the users.

Table 2 shows our system’s performance, in the row “new system.” The next rows show different experiment settings discussed in the next two sections. As a baseline, we evaluated the performance of the widely used FKGL readability formula. The average agreement between this formula and the AMT annotators was .531. Our system achieved an agreement of .734 with the AMT annotators, outperforming the FKGL baseline by 38.3%. The increase is statistically significant as assessed by a Wilcoxon signed rank test at the *P*=.05 level.

We also trained and tested separate models for each of the disease topics following the same process. Our system showed consistent improvement over the baseline across all disease categories. Agreement in the diabetes and hypertension categories increased significantly over the baseline FKGL metric. The cancer category improved substantially, but not significantly, over the baseline. These results suggested that our method is robust across different topics.

### User Behavior

**Figure 2:**
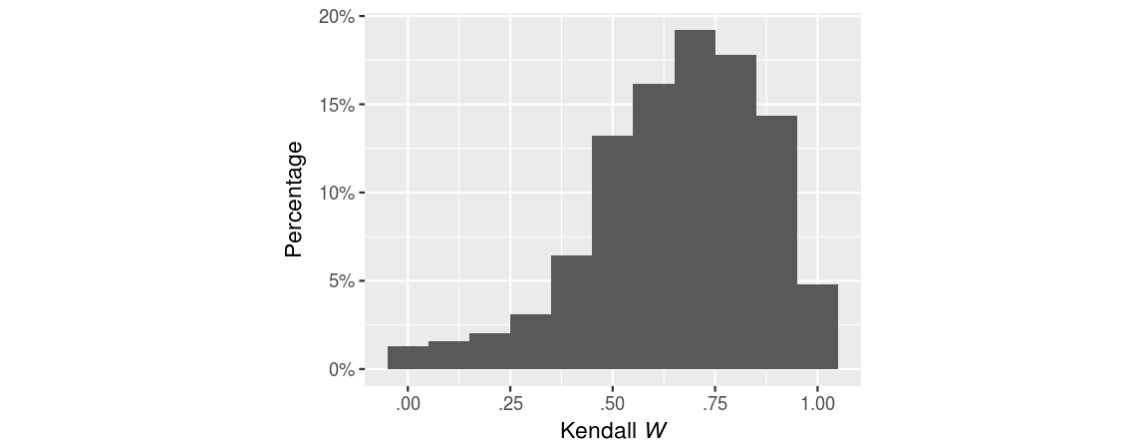
Histogram of Kendall *W* evaluating readability ratings between any 2 Amazon Mechanical Turk users.

A variety of factors may influence a reader’s reading comprehension, which in turn determines his or her judgment on a document’s difficulty. We examined the differences in the AMT users’ difficulty ratings using the same Kendall *W* coefficient. We calculated *W* for each pair of users’ ranked documents. The average concordance between any 2 users was .658. Figure 2 shows the distribution of concordance between any 2 users in our dataset.

While there are pairs of users whose concordance was low, most (851/1299, 65.51%) had a concordance greater than .6. When examined on an individual level, the low concordance can often be attributed to a few users who appeared to disagree with many others. There were 9 users who had a less than .5 concordance with more than 10 other users. Furthermore, 5 of these users’ mean concordance with other users was less than .5.

**Table 2:** System performance (Kendall *W*) compared with baseline for specific disease topics and with partial datasets. Numbers in parentheses are percentage improvements over FKGL (Flesch-Kincaid Grade Level). *P* values are comparisons with FKGL using a Wilcoxon signed rank test.

System		Cancer		Diabetes		Hypertension		All	
System		Kendall *W*	*P* value	Kendall *W*	*P* value	Kendall *W*	*P* value	Kendall *W*	*P* value
FKGL (baseline)		.541		.490		.561		.531	
New system		.656 (+21.3)	.08	.790 (+61.3)	.02	.715 (+27.5)	.03	.734 (+38.3)	<.001
**New system with data subsets excluded**					
	Excluding eccentric users	.694 (+28.3)	.03	.762 (+55.5)	.02	.727 (+29.6)	.03	.722 (+36.0)	<.001
	Excluding controversial documents	.650 (+20.1)	.05	.790 (+61.3)	.02	.759 (+35.2)	.02	.737 (+39.0)	<.01

**Figure 3:**
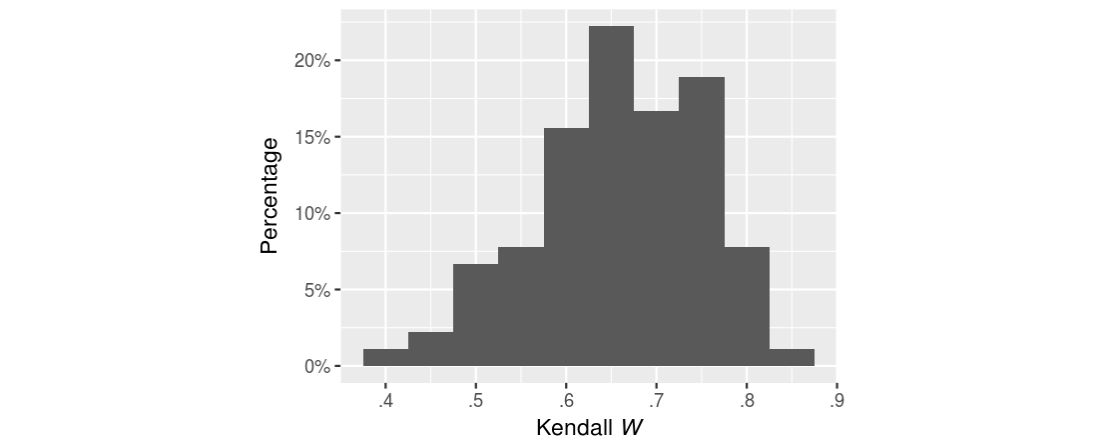
Histogram of individual Amazon Mechanical Turk users' conformity (measured by the mean of Kendall *W* against their peers).

To measure a user’s conformity in relation to others, we calculated the mean Kendall *W* between individual users and all of their peers. Figure 3 shows the distribution.

Approximately one-third of the users were highly conforming (mean *W≥*.7) with others, whereas 7% (6/90) were eccentric (mean *W*<.5). This result suggests that, despite individual differences in their background knowledge about the subject matter, AMT users still exhibited a consensus on a document’s difficulty level. We also noted that our system was able to predict readability orders similar to those of a “regular” user. Our system’s mean *W* was highly correlated with a user’s conformity (ρ=.85). In contrast, the FKGL formula’s predicted grade levels did not show a strong correlation (ρ=–.13) with conformity.

Table 2 (row “–eccentric users”) shows the performance of models trained from data excluding eccentric users. All disease topics performed significantly better with our system than with FKGL. Our system’s performance on the combined disease topics, also significantly higher than with FKGL, was slightly lower than with the system using the full dataset. This could be due to the large amount of samples removed from training even when we excluded only a small number of users, because the difference vectors were generated from all possible pairwise comparisons. On the individual disease topic level, however, the cancer and hypertension models outperformed our system when trained on the full training data.

### Controversial Documents

**Figure 4:**
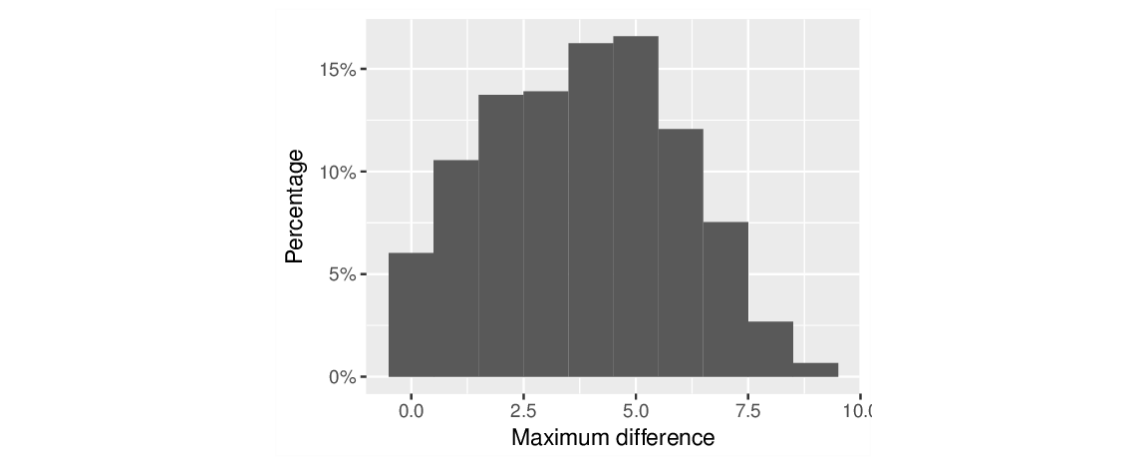
Histogram of maximum differences in Amazon Mechanical Turk users' ratings of documents rated by at least two users.

In addition to annotator differences, another factor that contributes to inconsistent annotations is the nature of the documents. We postulated that some documents may have been challenging for the AMT users. For example, certain types of domain-specific writing may appear easy to understand to some but not all users, leading to inconsistent user ratings. These “controversial” documents would also have confused our system, which attempted to learn from the conflicting human annotation. To highlight the range of AMT users’ perceptions of difficulty, Figure 4 shows the maximum difference in ratings assigned by AMT users to documents that were rated by at least two users (n=597).

The mean difference was 3.8, suggesting that users’ perceptions of difficulty varied considerably. The 2 sources of documents (Wikipedia and EHR notes) contained approximately the same number of controversial documents (maximum difference >5), and the cancer topic had more such documents than the other 2 topics. We further trained new models after removing controversial documents from the dataset. Table 2 shows the performances of these models in the last row (“Excluding controversial documents”). Performance of 2 categories, cancer and diabetes, remained similar to those of the models trained from the full dataset. The hypertension set increased appreciably.

### Feature Ablation

We compared the contribution of the different types of features included in our system. We trained separate models without the word frequency–based features, readability formula features, word length–based features, and word embedding–based features. Table 3 shows the performance of these models.

Excluding word embeddings resulted in the largest decrease in performance. The word frequency–based features did not appear to contribute much to the overall performance. Removing these features resulted in only a 0.1% performance decrease. This could be due to the nature of the word frequency corpus (a general English corpus without any particular emphasis on any domain) we used to calculate these features. The surface text characteristics captured by the formulas showed a moderate contribution, although they were not reliable stand-alone indicators. With the exception of 1 case, the contributions of the features were consistent across different disease topics—word embedding and word length–based features being the highest and word frequency the lowest.

**Table 3:** Model performance (Kendall *W*) with feature ablation.

Feature set		Cancer	Diabetes	Hypertension	All
Full^a^		.656	.790	.715	.734
**Excluded feature**					
	Frequency	.652	.792	.710	.733
	Formula	.648	.789	.709	.728
	Length	.636	.785	.702	.716
	Embedding	.677	.784	.703	.714

^a^The system with all proposed features included (data from Table 2).

## Discussion

### Principal Findings

We explored methods to automatically assess the readability levels of clinical narratives. Our ranking-based system using simple textual features and easy-to-learn word embeddings outperformed predictions from applying FKGL. In all of the disease topics we assessed, our method achieved an over 20% increase, with the majority of cases showing higher and statistically significance increases.

### Limitations

One limitation of our method is that it may be necessary to prune inconsistent data before training a model. Some users’ perceptions of document readability may exhibit a different pattern from others’. Including conflicting data points may result in suboptimal models. A future study direction is to explore the trade-off between expert and crowdsourced annotations.

Another limitation is that we trained our model on AMT users’ perceived document difficulty, which can be different from a linguistic perspective.

### Comparison With Other Methods

We applied a learning-to-rank approach to readability assessment, whereby we used comparisons of relative difficulty to train a model and, similarly, to predict an order based on document difficulty. Existing machine learning–based systems are usually designed around classification. They are often limited to a few predefined labels [15] or require corpora labeled at distinct levels [14]. The advantage of our approach is that we do not need expert annotation of grade levels on documents, and annotation may be crowdsourced as in our experiments. Acquiring more personalized training examples is also possible without explicit curation, as user actions may be implicitly mined to generate document difficulty comparisons, by using information retrieval methods.

Furthermore, unlike many other machine learning–based methods that require deep natural language processing, such as parsing [28] and discourse analysis [29], our choice of feature set is relatively simple. The surface features from readability formulas and word frequencies were both easy to calculate. Well-established tools also exist to generate word embeddings from large corpora. Therefore, our system could be easily deployed in an EHR system.

Lastly, although traditional readability formulas are very easy to use by nontechnical users, as they do not require training a machine learning model, they are inaccurate in determining the difficulty of complex documents. With simple features and widely available software packages, our proposed method is straightforward to implement.

### Conclusions

Patients’ access to their EHR notes has increased dramatically according to US national statistics. However, actively engaging patients in the management of their own health remains challenging. Assessing the readability of EHR notes and integrating educational assistance may make these notes more accessible for a layperson without professional training in medicine. To this end, we developed a new machine learning–based method to assess EHR readability from relative orders of text difficulty. We trained a learning-to-rank system to predict relative difficulty levels of given documents, instead of using the traditional classification approach, in which documents are assigned levels from a limited predefined set of values. Our experiments showed that this method significantly outperformed the widely used FKGL formula, and the improvement was consistent across different topics. Our system’s average concordance with an individual human user’s ratings was higher than the concordance between different human annotators. This method can potentially be personalized to individual users to better accommodate their background knowledge.

## Abbreviations

AMT: Amazon Mechanical Turk
EHR: electronic health record
FKGL: Flesch-Kincaid Grade Level
SVM: support vector machine

## References

[1] Greene J, Hibbard JH (2012). Why does patient activation matter? An examination of the relationships between patient activation and health-related outcomes. J Gen Intern Med.

[2] Begum N, Donald M, Ozolins IZ, Dower J (2011). Hospital admissions, emergency department utilisation and patient activation for self-management among people with diabetes. Diabetes Res Clin Pract.

[3] Hibbard JH, Greene J (2013). What the evidence shows about patient activation: better health outcomes and care experiences; fewer data on costs. Health Aff (Millwood).

[4] Mosen DM, Schmittdiel J, Hibbard J, Sobel D, Remmers C, Bellows J (2007). Is patient activation associated with outcomes of care for adults with chronic conditions?. J Ambul Care Manage.

[5] Henry J, Pylypchuk Y, Patel V (2016). Electronic capabilities for patients among U.S. non-federal acute care hospitals: 2012-2015. ONC data brief 38.

[6] Agarwal N, Hansberry DR, Sabourin V, Tomei KL, Prestigiacomo CJ (2013). A comparative analysis of the quality of patient education materials from medical specialties. JAMA Intern Med.

[7] Huang G, Fang CH, Agarwal N, Bhagat N, Eloy JA, Langer PD (2015). Assessment of online patient education materials from major ophthalmologic associations. JAMA Ophthalmol.

[8] Watad A, Bragazzi NL, Brigo F, Sharif K, Amital H, McGonagle D, Shoenfeld Y, Adawi M (2017). Readability of Wikipedia pages on autoimmune disorders: systematic quantitative assessment. J Med Internet Res.

[9] Brigo F, Otte WM, Igwe SC, Tezzon F, Nardone R (2015). Clearly written, easily comprehended? The readability of websites providing information on epilepsy. Epilepsy Behav.

[10] Brigo F, Erro R (2015). The readability of the English Wikipedia article on Parkinson's disease. Neurol Sci.

[11] Davis GT, Singh H (2011). Should patients get direct access to their laboratory test results? An answer with many questions. JAMA.

[12] Koh HK, Brach C, Harris LM, Parchman ML (2013). A proposed 'health literate care model' would constitute a systems approach to improving patients' engagement in care. Health Aff (Millwood).

[13] Flesch R (1948). A new readability yardstick. J Appl Psychol.

[14] Kim H, Goryachev S, Rosemblat G, Browne A, Keselman A, Zeng-Treitler Q (2007). Beyond surface characteristics: a new health text-specific readability measurement. AMIA Annu Symp Proc.

[15] Leroy G, Miller T, Rosemblat G, Browne A (2008). A balanced approach to health information evaluation: a vocabulary-based naïve Bayes classifier and readability formulas. J Am Soc Inf Sci.

[16] Redish J (2000). Readability formulas have even more limitations than Klare discusses. ACM J Comput Doc.

[17] O'Bryant SE, Lucas JA, Willis FB, Smith GE, Graff-Radford NR, Ivnik RJ (2007). Discrepancies between self-reported years of education and estimated reading level among elderly community-dwelling African-Americans: analysis of the MOAANS data. Arch Clin Neuropsychol.

[18] Manly JJ, Jacobs DM, Touradji P, Small SA, Stern Y (2002). Reading level attenuates differences in neuropsychological test performance between African American and white elders. J Int Neuropsychol Soc.

[19] Manly JJ, Schupf N, Tang M, Stern Y (2005). Cognitive decline and literacy among ethnically diverse elders. J Geriatr Psychiatry Neurol.

[20] Zheng J, Yu H (2018). Ranking readability demo.

[21] Keselman A, Slaughter L, Smith CA, Kim H, Divita G, Browne A, Tsai C, Zeng-Treitler Q (2007). Towards consumer-friendly PHRs: patients' experience with reviewing their health records. AMIA Annu Symp Proc.

[22] Pyper C, Amery J, Watson M, Crook C (2004). Patients' experiences when accessing their on-line electronic patient records in primary care. Br J Gen Pract.

[23] Joachims T (2006). Training linear SVMs in linear time.

[24] Zheng J, Yu H (2017). Readability formulas and user perceptions of electronic health records difficulty: a corpus study. J Med Internet Res.

[25] Leroy G, Kauchak D (2014). The effect of word familiarity on actual and perceived text difficulty. J Am Med Inform Assoc.

[26] Mikolov T, Chen K, Corrado G, Dean J (2013). Efficient estimation of word representations in vector space.

[27] Kendall MG, Smith BB (1939). The problem of m rankings. Ann Math Stat.

[28] Schwarm S, Ostendorf M (2005). Reading level assessment using support vector machines and statistical language models.

[29] Feng L, Jansche M, Huenerfauth M, Elhadad N (2010). A comparison of features for automatic readability assessment.

